# Photodynamic therapy induced cell cycle arrest and cancer cell synchronization: review

**DOI:** 10.3389/fonc.2023.1225694

**Published:** 2023-07-12

**Authors:** Kave Moloudi, Heidi Abrahamse, Blassan P. George

**Affiliations:** Laser Research Centre (LRC) Faculty of Health Sciences, University of Johannesburg, Johannesburg, South Africa

**Keywords:** photodynamic therapy, cell cycle arrest, mathematical models, photosensitizer, cancer therapy, ROS

## Abstract

Cell cycle arrest (CCA) is seen as a prime candidate for effective cancer therapy. This mechanism can help researchers to create new treatments to target cancer cells at particular stages of the cell cycle (CC). The CCA is a characteristic of various therapeutic modalities, including radiation (RT) and chemotherapy (CT), which synchronizes the cells and facilitates the standardization of radio-chemotherapy protocols. Although it was discovered that photodynamic treatment (PDT) had a biological effect on CCA in cancer cells, the mechanism remains unclear. Furthermore, besides conventional forms of cell death such as apoptosis, autophagy, and necrosis, various unconventional types of cell death including pyroptosis, mitotic catastrophe, paraptosis, ferroptosis, necroptosis, and parthanatos after PDT have been reported. Thus, a variety of elements, such as oxygen, the tumor’s microenvironment, the characteristics of light, and photosensitizer (PS), influence the effectiveness of the PDT treatment, which have not yet been studied clearly. This review focuses on CCA induced by PDT for a variety of PSs agents on various cell lines. The CCA by PDT can be viewed as a remarkable effect and instructive for the management of the PDT protocol. Regarding the relationship between the quantity of reactive oxygen species (ROS) and its biological consequences, we have proposed two mathematical models in PDT. Finally, we have gathered recent *in vitro* and *in vivo* studies about CCA post-PDT at various stages and made suggestions about how it can standardize, potentiate, and customize the PDT methodology.

## 1 Introduction

Photodynamic therapy (PDT) or light-activated treatment is increasingly being employed as adjuvant with other common modalities to treat cancer (1, 2). PDT is considered as a non-invasive strategy to treat various cancer types such as oral, skin, prostate, and head and neck (3, 4). Hence, PDT requires photosensitizer (PS) agent to be activated by light and generates numerous highly reactive molecular species including free radicals, reactive oxygen species (ROS), and singlet oxygen. However, PDT is beneficial and safe for the patient and the physician because it minimizes the interventional surgery, has short recovery periods, and protects organ integrity with a relatively low risk of local and systemic side effects (5–7). The response of PDT is based on the contribution of the three biophysical interactions between the PS, light of suitable wavelength, and oxygen molecules in the cells, which causes the specified effects within pathological tissues (8). In the presence of light, PS is activated and produces ROS. Afterward, ROS are responsible for damaging and killing tumor cells (9). Excitation of the PS with light leads to electron (e) and proton (p) movement to the first singlet excited state. Triplet mode is created after intersystem crossing. The triplet PS transfers energy to triplet oxygen, leading to the assembly and generation of reactive singlet oxygen (^1^O_2_). ^1^O_2_ can exert a multitude of actions including direct damaging of cancer cells, destroying the vascular system, and irritating the immune responses (10–13). From the literature on PDT studies, it can be concluded that ROS has various biological effects (14). In fact, it is now well established that PDT can cause tumor destruction by three mechanisms: direct cell death, tumor vascular damage, and immune response induction (15–17). It seems that the mechanisms of PDT in terms of cellular and molecular response requires further investigation. Photochemical reactions trigger various types of cell death mechanisms that lead to tumor tissue destruction, for instance, the shutdown of tumor vascular, leading to necrosis of the tumor via deprivation of oxygen and nutrients (18, 19). For many years, conventional cell death by PDT is divided into three types such as apoptosis, autophagy, and necrosis (20, 21). The molecular mechanisms of photo-killing pathways of PDT-induced apoptosis and autophagy ultimately cause low survival and cytotoxicity effects (22, 23). Moreover, in recent years, the striking progress in molecular techniques greatly expanded, resulting in new forms of cell death being discovered, such as mitotic catastrophe (MC), paraptosis, pyroptosis, parthanatos, necroptosis, and ferroptosis. Additionally, it has been reported that photodamages to the endoplasmic reticulum (ER) and liposomes result in apoptosis and paraptosis cell death (24, 25).

PDT may be more effective if it targets cancer cells at particular times of the cell cycle (CC). For example, targeting cancer cells in the S phase may increase the amount of PS uptake and ROS generation, leading to more efficient cell death via impeding the proliferation and cell cycle arrest (CCA) due to extensive DNA and organelle damages and mitotic and checkpoint failures. To date, various signs of CCA have been reported in cancerous cells under PDT that eventually cause cell damage, apoptosis, mitotic catastrophe, and cell death. Therefore, in this review, we focus on CCA in PDT and how it helps the standardization of PDT protocol and gives new insight to researchers to develop new therapies to target cancer cells at specific phases of the CC.

## 2 Mechanism of PDT

**Figure 1:**
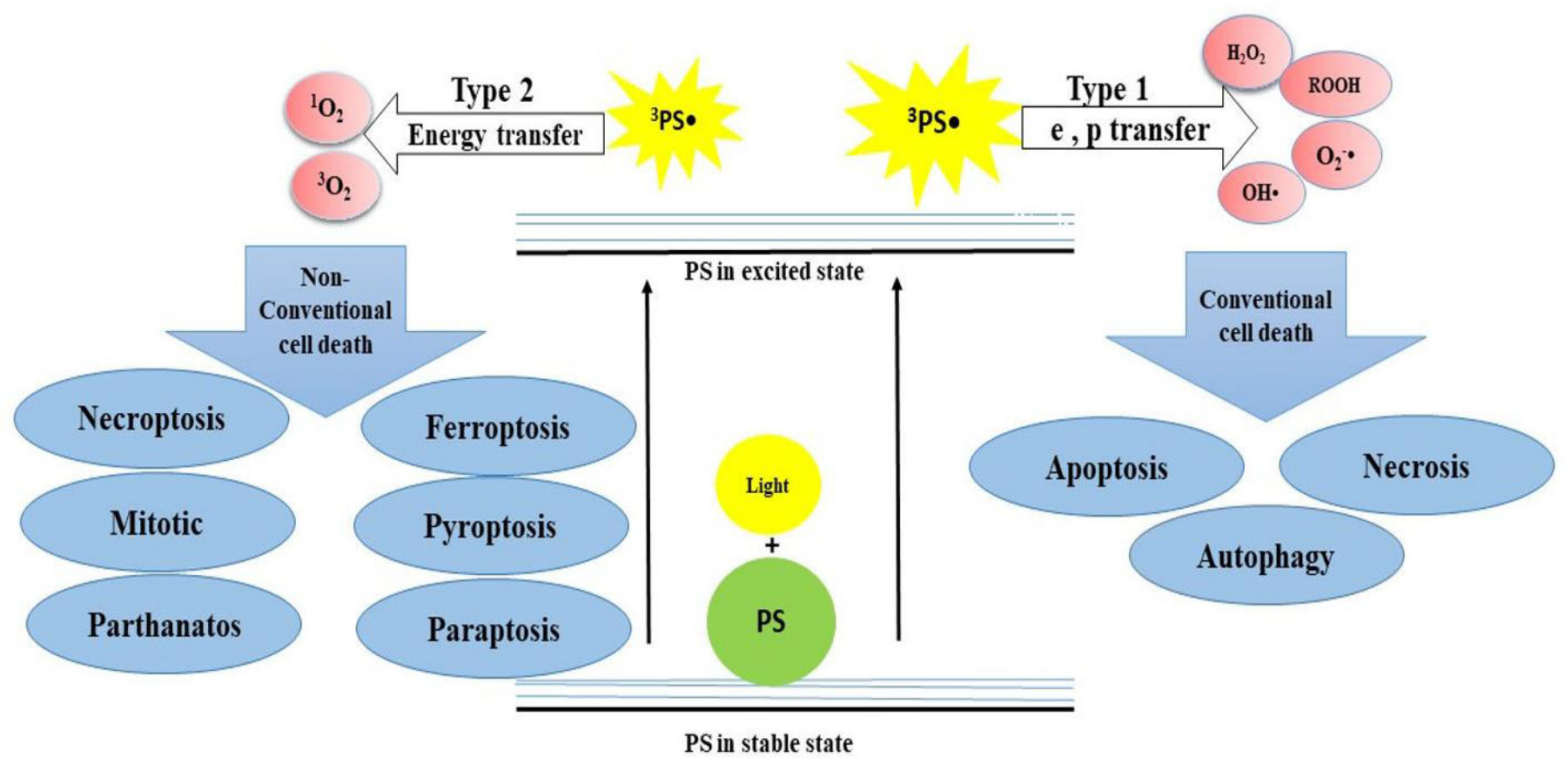
The mechanism of action of PDT that acts in two stages. (I) PSs absorb light and become excited to higher energy levels and (II) excited PSs are relaxed and transfer energy to the O_2_, leading to the generation of ROS, ^1^O_2_, and other free radicals.

PDT is a cancer treatment procedure, which employs PSs that are activated under light exposure of certain wavelengths. PDT acts in two stages (**Figure 1**): (1) PSs absorb light and become excited to higher energy levels, and (2) excited PSs are relaxed and transfer energy to the oxygen molecules, resulting in the generation of ROS, ^1^O_2_, and other free radicals. This can cause damage to DNA, membrane, and other organelles in cells in two ways. In type I, ^3^PS• transfers electrons and proton from the nearby molecules such as polyunsaturated fatty acids (PUFAs) in the membrane of a cell. Consequently, the interaction of e and p with cellular oxygen leads to the generation of cytotoxic ROS such as superoxide anion (O_2_^−^•), hydroperoxide radical (HOO•), peroxides (H_2_O_2_, ROOH), and hydroxyl radical (OH•), which trigger free radical chain reactions. Type II involves the transfer of energy from ^3^PS to molecular oxygen, resulting in the formation of ^1^O_2_, which is considered a powerful oxidizing agent. Both type I and II reactions can happen simultaneously, and the ratio between them depends mainly on the photo-chemical and photo-physical features of the PS and cellular oxygen. Finally, these reactions trigger various cell death mechanisms in cancer cells in different pathways and lead to the suppression of tumor tissue (19, 26) (**Figure 1**). Depending on the site of damage, cell death occurs through apoptosis, necrosis, or autophagy. However, several important parameters can influence the PDT performance, including PS properties, the wavelength of light, and amount of oxygen in cells (27).

## 3 CC in normal vs. cancer cells

The normal CC includes G1, S, G2, and M phases. During the G1 phase, the cell grows and prepares to enter the S phase, where DNA replication occurs, resulting in two identical copies of the cells’ DNA. The G2 phase is a period of growth and preparation for cell division. Finally, during the M phase, the cell divides into two same cells through a process called mitosis. After the M phase, the cells may enter a resting state called the G0 phase, where they remain until stimulated to re-enter the CC. Each phase is depending on the type of cell and external factors such as growth factors and stress signals (28). Cyclins and cyclin-dependent kinases (CDKs) are key regulatory proteins that control the progression of cells through the CC. Cyclin D is synthesized during the G1 phase, which binds to CDK4/6 to form an active complex. This complex phosphorylates the retinoblastoma (Rb) protein, which releases transcription factors that drive the expression of genes required for DNA synthesis. In the S phase, DNA replication occurs, and cyclin E is synthesized, which binds to CDK2 to form an active complex. Then, this complex phosphorylates proteins involved in DNA replication and repair. In addition, transforming growth factor beta (TGFβ) can activate cyclin E and CDK2 complex. In the G2 phase, the cell prepares for mitosis by synthesizing various proteins required for cell division. Cyclin A is synthesized during this phase, which binds to CDK1 to form an active complex. This complex drives the cell into mitosis by phosphorylating proteins involved in chromosome condensation and spindle formation. Overall, the regulation of cyclins and CDKs is essential for the progression of CC and genomic stability. Dysregulation of these proteins can lead to uncontrolled cell proliferation and tumor formation (29, 30).

**Figure 2:**
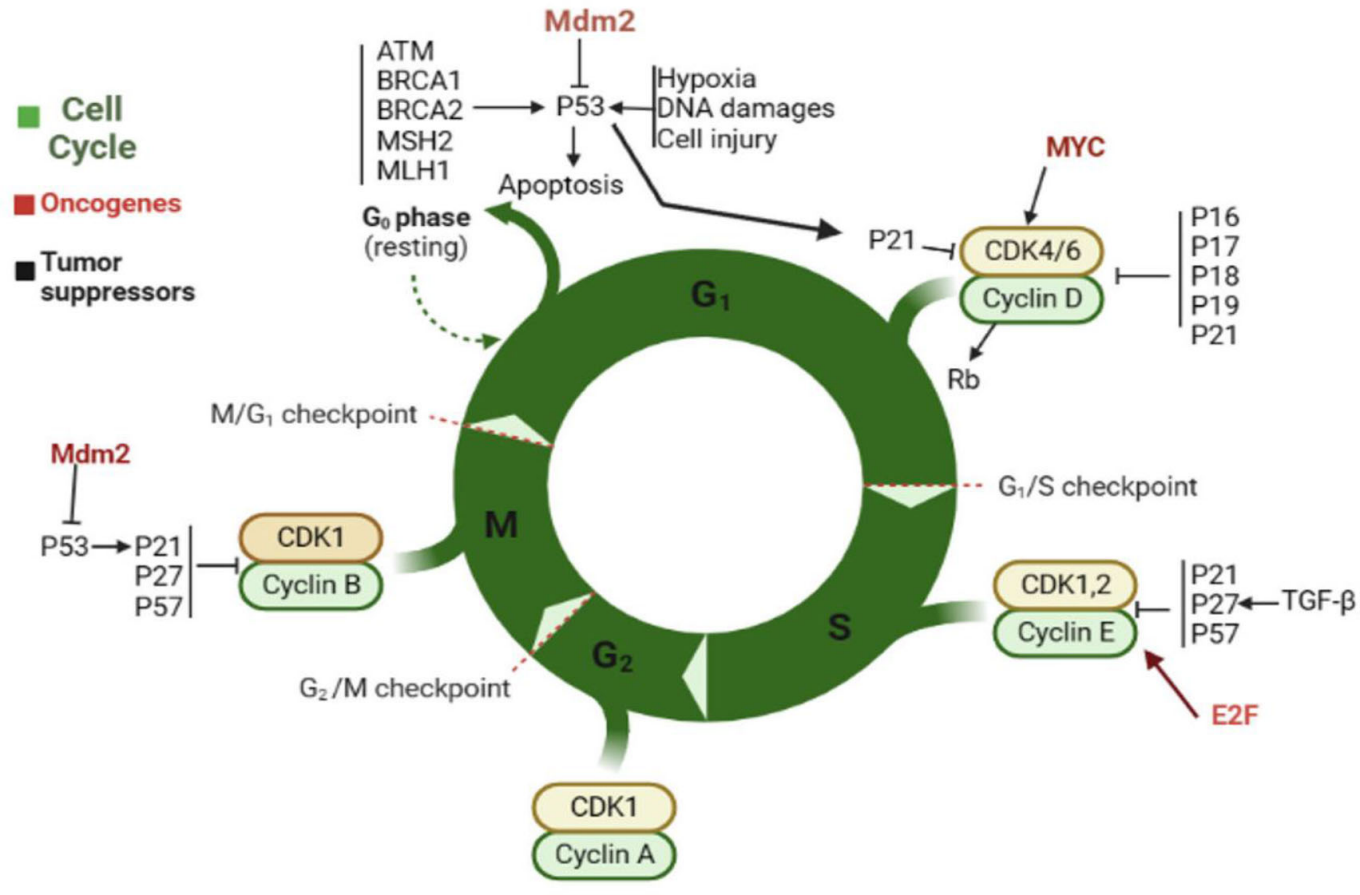
The normal CC and factors that contribute to cell progression. Oncogenes have been highlighted in brown.

In cancer cells, the CC is often disrupted and uncontrolled, leading to abnormal growth and division. Cancer cells can divide rapidly and continuously without the normal checkpoints that regulate the CC in healthy cells. In some cases, cancer cells may skip the G1 phase and enter directly into DNA replication, leading to an increase in the number of abnormal cells. Additionally, cancer cells may have mutations in genes (cyclins) and oncogenes such as Mdm2, MYC, and E2F that lead to uncontrolled growth and division. As a result of these disruptions, cancer cells may continue to divide and grow, even in the absence of external signals. This uncontrolled growth can lead to the formation of tumors and the spread of cancer. As shown in **Figure 2**, in normal cells, several genes control the CC, including the following. First is P53: this gene produces p53 protein to suppress tumor cells by regulating the G1 checkpoint of the CC or promoting apoptosis. Additionally, it aids in preventing the replication and potential mutation-causing effects of cells with damaged DNA. Some proteins such as ATM, BRCA1, 2, MSH2, and MLH1, and the cell conditional such as hypoxia and cell damage cause P53 activation. Second is the RB1: this gene produces a protein called Rb, which helps to regulate the G1 checkpoint. It prevents the activity of E2F transcription factors, preventing them from promoting cell division. Third is the CDK2: this gene produces two proteins, p16 and p14, which also act as tumor suppressors by regulating the G1 checkpoint. Moreover, other proteins such as p17, p18, p19, and p21 play a critical role in this stage. They inhibit the activity of CDK4/6, preventing the phosphorylation of Rb and promoting CCA (31, 32).

The cancer CC is uncontrolled, and this characteristic is crucial for the survival of cancer cells. Unlike normal cells, they undergo cell division only when necessary for growth or repair; cancer cells divide uncontrollably, leading to the formation of tumors. In the G1 phase, the cell grows and prepares for DNA replication. In the S phase, DNA replication occurs, resulting in the formation of two identical copies of DNA. In the G2 phase, essential proteins are synthesized, and the cell prepares for mitosis. Eventually, the cell divides into two daughter cells during the M phase (33–35). However, this paradigm highlights the critical role of checkpoints in cancer to provide a better comprehensive CC control and specific checkpoint functions and create new therapeutic opportunities. Protein kinases are enzymes that play a vital role in regulating the CC checkpoints. They work by adding phosphate groups to other proteins, which can either activate or prevent their function. In the context of CC checkpoints, protein kinases act as gatekeepers that ensure that the cell is ready to proceed to the next phase (36, 37).

## 4 CC phases

**Figure 3:**
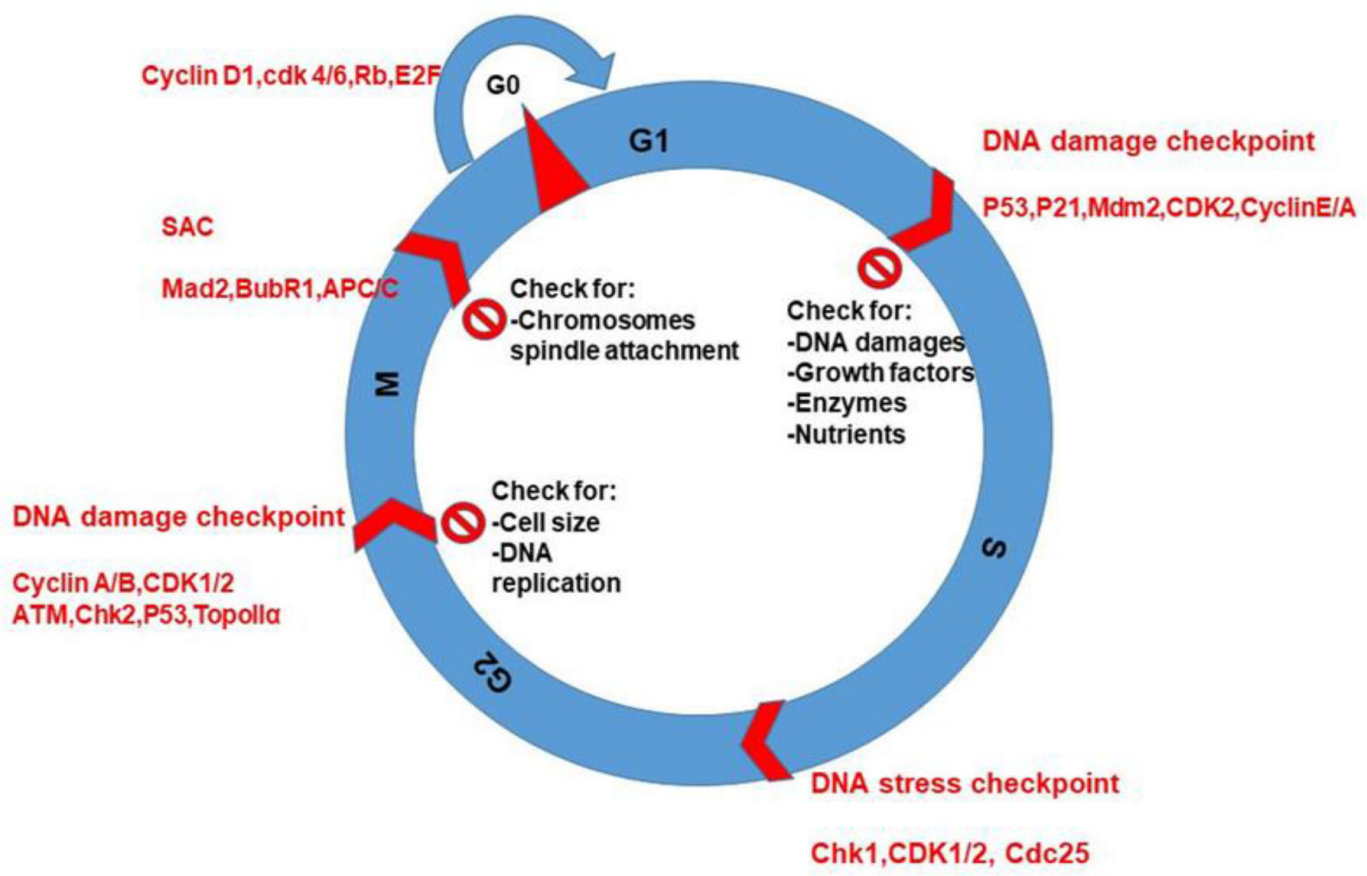
CC phases, checkpoints protein kinases, and cyclins during cell cycling. The purpose of checking is for DNA replication and damages, nutrients, and spindle attachment to chromosomes during CC.

Similar to normal cells, cancer cells have four phases in CC such as G1, S, G2, and M (38). Cancer cells divide more rapidly than normal cells and may have mutations that allow them to bypass normal CC checkpoints. This uncontrolled and abnormal cell division leads to the formation of tumors (39, 40). However, all CC phases are important to regulate and progress the CC during G1 and chromosome segregation during M phase (**Figure 3**).

### 4.1 CC entry and progression

CC entry and progression are tightly regulated processes in normal cells. External signals and growth factors activate signaling pathways that promote the entry of cells into the CC. Once cells are in the CC, checkpoints ensure that cells progress through each phase only when conditions are favorable (**Figure 3**). The movement from G1 to S phase is controlled by the activity of CDKs and their regulatory subunits, cyclins. These proteins interact to form complexes that phosphorylate key targets, allowing to progress of the CC (41, 42). The G1 checkpoint ensures that the cell has sufficient nutrients, oxygen, and energy to enter S phase. If the cell fails to meet these requirements, it will remain in G1 and not progress through the CC. Sometimes, due to deprivation of oxygen and energy, cells bypass progression and stay in G0. The G1 checkpoint is regulated by tumor suppressor genes such as p53, which can activate CCA or apoptosis if DNA damage is detected. During S phase, DNA replication occurs, and all chromosomes should be replicated correctly before progressing to G2. The S phase checkpoint monitors DNA replication and can arrest the CC if errors are detected. In G2, the cell prepares for mitosis by producing essential proteins. The G2 checkpoint ensures that DNA replication is complete without any errors. The progression through mitosis is regulated by CDKs and cyclins, which control the assembly and disassembly of the mitotic spindle and the segregation of chromosomes (36, 43). Overall, the regulation of the CC is critical for maintaining proper cell growth and preventing errors that can lead to cancer. Understanding these mechanisms can help researchers develop new therapies to target cancer cells at specific phases of the CC. Cyclins are a family of proteins that play a critical role in regulating the CC. They bind to and activate CDKs, which promote the transition from one phase to the next (44).

### 4.2 CC checkpoints

The checkpoints are critical control mechanisms that ensure the proper progression of cells through the various stages. These checkpoints are responsible for detecting and correcting errors that may occur during DNA replication or cell division, ensuring that the resulting daughter cells are healthy and genetically stable. These checkpoints depend on conservative and evaluation signaling pathways that monitor DNA damage during all four phases of CC (**Figure 3**).

#### 4.2.1 DNA damage checkpoint

The DNA damage checkpoint is a specific type of checkpoint that occurs in response to DNA damage. This checkpoint can occur at any stage of the CC and is responsible for detecting and repairing DNA damage before the cell proceeds with replication or division. There are several proteins involved in the DNA damage checkpoint, including ataxia telangectasia mutated (ATM), ChK1/2, P53, and Topollα. These proteins detect and respond to DNA damage by phosphorylating downstream targets that activate repair mechanisms or induce cell CCA. Throughout the interphase, double-strand breaks (DSBs) of DNA trigger a rapid signaling response that depends on the checkpoint protein kinase (ATM), leading to the CCA and preventing to forward movement (45, 46). When DNA damage is detected, the checkpoint activates a signaling cascade that leads to the activation of repair mechanisms or, in severe cases, the induction of cell death. This checkpoint is crucial for preventing the accumulation of mutations and maintaining genomic stability. The DNA damage sensor complex MRN (Mre1, Rad50, and Nbs1) actives ATM and phosphorylates a few numbers of substrates (47, 48), but critical targets are the Chk2 and the p53 for the progression of the CC (49–51). The p53 activates the CDK inhibitor p21, leading to the inhibition of cyclin–CDK complexes mainly in G1 to prevent S phase entry. In the S and G2 phases, Chk2 degrades Cdc25 proteins, while p53 and ATM are not as critical during S and G2 phases (51).

#### 4.2.2 DNA replication stress checkpoint

The DNA replication stress checkpoint is active during the S phase only. Some obstacles such as ssDNA and stress obstruct DNA replication for successful duplication (52). Many exogenous (radiation, air pollution, and diet) and endogenous (inflammation and cytokines) factors under normal physiological conditions can cause DNA replication stress in cells (53, 54). The checkpoint involves a complex network of signaling pathways that coordinate the activation of DNA repair mechanisms, CCA, and apoptosis to prevent the propagation of damaged DNA. The key players in this checkpoint are the ATR (ataxia-telangiectasia) and Rad3-related kinases and their downstream effectors, such as CHK1 (checkpoint kinase 1) and p53 (**Figure 3**) (55, 56). DNA replication stress does not damage but activates the DNA replication checkpoint to prevent replication and the problem be solved. Consequently, an important part of the response is delay in mitotic entry and spending more time for replication to be correct. The checkpoint controls CC progression by restricting CDK activity, primarily through Chk1-dependent phosphorylation of Cdc25, which results in its proteasomal degradation (57), and WEE1 gene, by promoting 14-3-3 binding (58, 59). When the replication machinery encounters an obstacle, ATR is recruited to the site of damage, where it phosphorylates CHK1 and other substrates. This leads to the activation of DNA repair pathways, such as homologous recombination or non-homologous end joining, to fix the damage before replication can proceed (58, 60–64). This prevents the initiation of replication at new sites, limits the overall rate of replication, and allows replication forks to recover and resume once the impediments are dealt with, ensuring that all areas of the genome are replicated (65–68).

#### 4.2.3 Spindle assembly checkpoint

The spindle assembly checkpoint (SAC) is a cellular mechanism that ensures accurate chromosome segregation during cell division. It monitors the attachment of chromosomes to the spindle microtubules. This action could be performed by Aurora B and CDK (69–71). The checkpoint involves the recruitment of several proteins, including MAD1, MAD2, BUB1, and BUBR1, to the kinetochores of the chromosomes. These proteins form a complex that monitors the tension and alignment of the chromosomes on the spindle (72–75). Once all kinetochores are attached and bi-oriented, a lack of SAC activity leads to the disassembly of the MCC, freeing up Cdc20 to act as a co-activator of APC/C (76, 77). If the chromosomes are not properly aligned or attached to the spindle, the checkpoint signals to the cell to delay the onset of anaphase, which is the stage of cell division that the chromosomes are separated. This delay allows time for the spindle apparatus to be properly formed or for the attachment of the chromosomes to be corrected (78, 79). If the problem remains unsolved, the cells follow two paths: either apoptosis via caspase activation (80) or slippage where cells exit the M phase without chromosome segregation and enter the next CC as a single tetraploid cell (81). Mitotic slippage occurs because of basal levels of cyclin B degradation during metaphase, eventually lowering the CDK1 activity to below the threshold levels for M phase exit (82). Overall, the spindle assembly checkpoint is a critical mechanism that ensures the accurate segregation of chromosomes during cell division and prevents the formation of aneuploid cells, which can lead to genetic disorders and cancer (83, 84).

## 5 CC control as cancer therapy target

Cancer cells often have defects in their DNA repair pathways, which allows them to accumulate mutations and grow uncontrollably. Hence, targeting these pathways could be a strategy for cancer therapy. One approach is to use DNA-damaging agents, such as CT or radiation, which induce DNA lesions that cancer cells cannot repair efficiently (85). These modalities remain the most effective for cancer therapy but also induce DNA damage in normal tissue (86). Another approach is to target specific DNA repair enzymes or proteins that are overexpressed or mutated in cancer cells, thereby disrupting their ability to repair DNA damage. In addition to targeting DNA repair pathways directly, cancer therapy also involves regulating the CC, which is tightly controlled to ensure proper DNA replication and segregation. Dysregulation of the CC is a hallmark of cancer, as cancer cells often bypass checkpoints that would normally trigger cell death or repair mechanisms. Therefore, drugs that inhibit specific CC regulators, such as CDKs, have been developed for cancer therapy.

**Figure 4:**
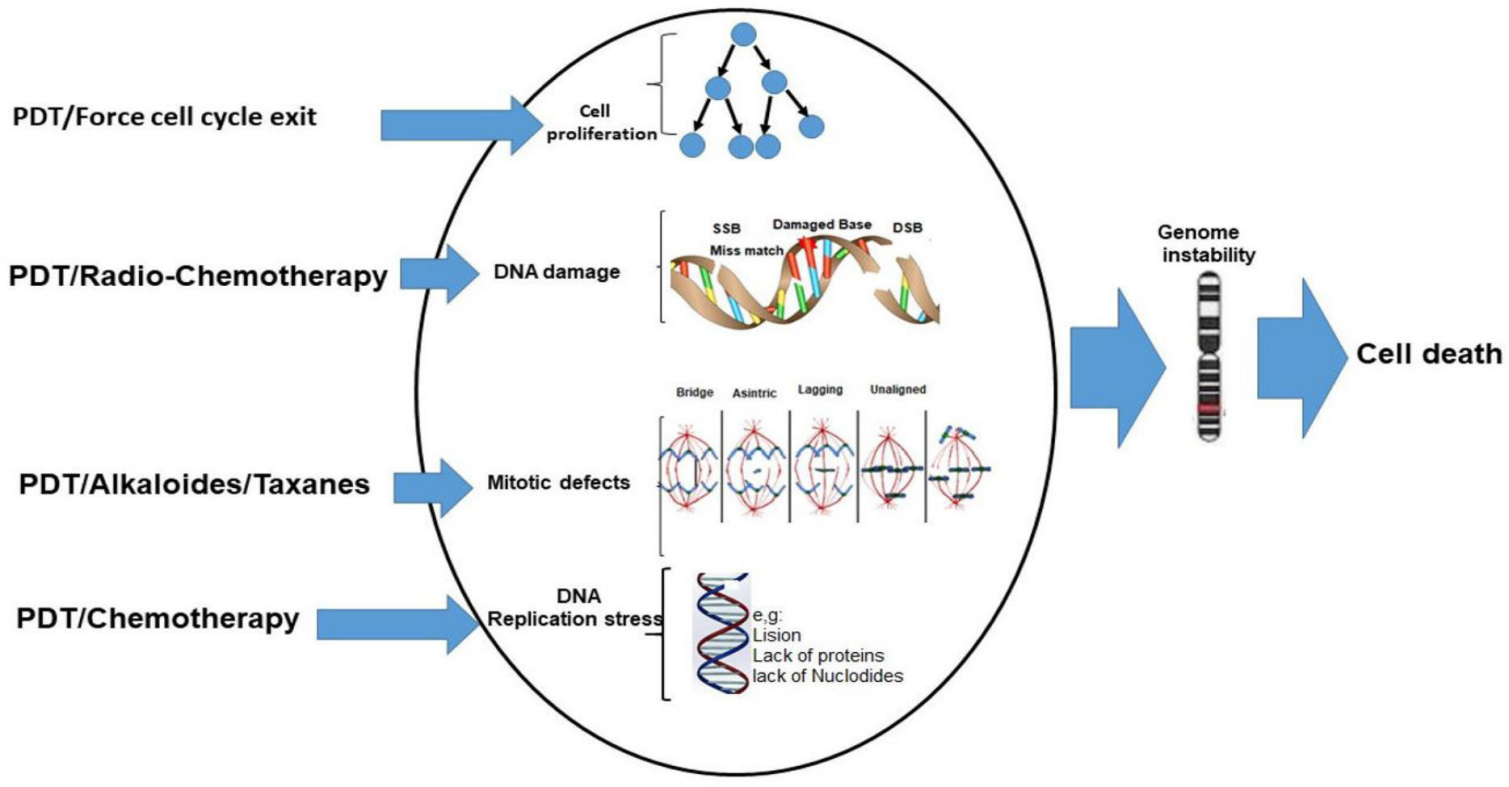
PDT in combination with other modalities such as RT, RT-CT, alkaloids, and taxanes in cancer therapy.

One promising approach for cancer therapy is the combination of PDT with RT or CT. When combined with RT or CT, PDT can enhance the effectiveness of these treatments by increasing DNA damage and inhibiting DNA repair pathways (87, 88) (**Figure 4**). Research has shown that PDT in combination with RT or CT can improve outcomes in various types of cancers, including lung, head and neck, and pancreas (89–91). This approach has also been shown to reduce side effects and toxicity associated with traditional cancer treatments. However, more research is needed to optimize the timing and dosing of these therapies and to identify biomarkers that can predict response to the treatments. Additionally, the development of more targeted photosensitizing agents and combination therapies tailored to individual patient’s genetic profiles may further improve outcomes in cancer therapy.

Alkaloids and taxanes are classes of CT drugs that have been used in combination with PDT for cancer treatment. Alkaloids, such as vinblastine and vincristine, act by disrupting the cell division process in cancer cells (92–95). Taxanes, such as paclitaxel and docetaxel, also interfere with cell division by stabilizing microtubules (96–98). These CT medications have the ability to improve the efficacy of PDT by boosting the production of ROS and reducing DNA repair pathways. This strategy has shown effective outcomes for breast, ovarian, and prostate cancer. However, as with any cancer treatment, there are potential side effects and toxicity associated with these drugs. Further research is needed to optimize the dosing and timing of these therapies and to identify biomarkers that can predict response to treatment. Additionally, the development of more targeted CT drugs and combination therapies tailored to individual patient’s genetic profiles may further improve outcomes in cancer therapy. Overall, understanding the complex interplay between DNA repair and CC control in cancer cells is crucial for developing effective therapies that can selectively target cancer cells with minimal side effects (**Figure 4**).

## 6 ROS production and its consequences in cells

**Figure 5:**
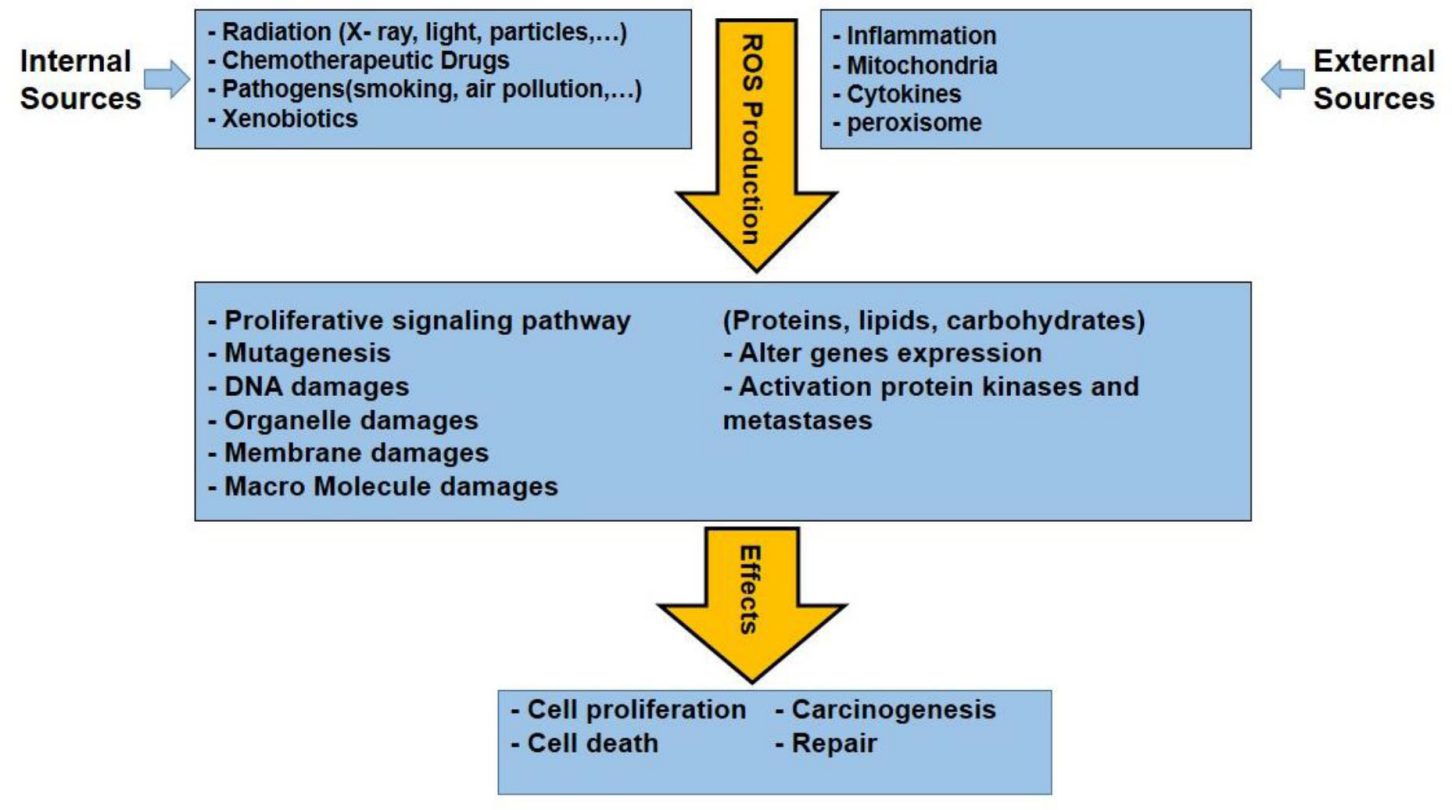
Various internal and external sources of ROS production and consequences effects of them that are leading some effects in cancerous cells.

ROS from both internal and external sources has an impact on biology (**Figure 5**). Radiation, chemotherapeutic medications, pathogenesis, xenobiotics, etc. are some examples of external causes, while the internal elements are including cytokines, inflammation, mitochondria, and peroxisomes (99–101).

Naturally, ROS are produced in cells as a byproduct of normal metabolic processes. ROS plays an important role in cellular signaling and defense against pathogens. In normal cells, ROS production is tightly regulated to maintain a balance between ROS generation and elimination by antioxidant systems. Uncontrolled ROS production induces oxidative stress, which can damage cellular components and contribute to the development of various diseases including cancer and neurodegenerative and cardiovascular diseases. In cancer, the intracellular ROS causes oxidation and mutations in pro-oncogenes such as Ras and p53 as tumor suppressor genes (102).

Indeed, ROS can have both beneficial and harmful (double-edged sword) effects on cells depending on their concentration and location. At low levels, ROS can act as signaling molecules to regulate cell proliferation, differentiation, and apoptosis. However, at high levels, ROS can cause oxidative damage in macromolecules such as lipids, proteins, and DNA, leading to cell death and tissue damage. Therefore, maintaining a balance in ROS levels is crucial for cellular homeostasis and overall health. The high level of ROS in mitochondria leads to the activation of mitogen-activated protein kinase (MAPK) and Ras-ERK. This signal induce cell proliferation, cell survival, cell migration, and epithelial–mesenchymal transition (103, 104).

Edderkaoui and co-workers reported that ROS generation in the extracellular matrix cause the survival of pancreatic cancer cells through 5-lipoxygenase (LOX) and NOX (105). For example, the combination of gemcitabine with trichostatin-A, epigallocate-3-gallate (EGCG), capsaicin, and benzylisothiocyanate (BITC) has been proven to be effective for pancreatic cancer treatment (106). All these drug action are based on the elevation of intracellular ROS levels to trigger apoptosis. Furthermore, it is reported nonsteroidal anti-inflammatory drugs (NSAIDs) like Sulindac enhances the intracellular level of ROS that causes cell death and apoptosis in colon and lung cancer cells that are more sensitive to H_2_O_2_ (107, 108). Similarly, an anticancer drug Aminoflavone promotes cell death in MCF-7 and MDA-MB-468 breast cancer cells. Aminoflavone induces programmed cell death due to an increased intracellular level of ROS and activation of caspase 3. Some clinical chemotherapeutic agents can cause cell death via mitochondrial DNA damage and increase cellular ROS levels (109, 110). The ROS production and its biological effects are summarized in **Figure 5**.

Chemotherapeutic agents such as doxorubicin, epirubicin, bleomycin, and platinum can also induce oxidative stress in cancer cells through the alteration of ROS levels. These drugs target mitochondria and generate ROS (111–113). For decades, RT has been utilized for cancer therapy. RT can induce DNA breaks and apoptosis to indirectly militate against the antitumor treatment by inducing the ROS levels (114, 115). PDT also induces the generation of various ROS, which prompts signaling cascades and cell death in cancer cells when exposed to light of specific wavelength (116, 117). Yokomizo et al. and Sasada et al. reported that thioredoxin could overcome cancer cell resistance via ROS-generating and its antioxidant activity (118, 119). However, Ravi et al. illustrated that thioredoxin in combination with daunomycin increases the cytotoxic effect in MCF-7 breast cancer cells. Daunomycin induces cancer cell death by redox cycling reactions and ROS generation. These processes cause DNA damage and apoptosis of tumor cells (**Figure 5**) (120, 121). Procarbazine, one of the earlier drugs, produces H_2_O_2_ via oxidation in an aqueous solution, which is crucial for cytotoxic activity. Subsequently, Mathe and colleagues have reported that procarbazine can be used for the treatment of Hodgkin’s lymphoma and hematolymphoid malignancies (122).

**Figure 6:**
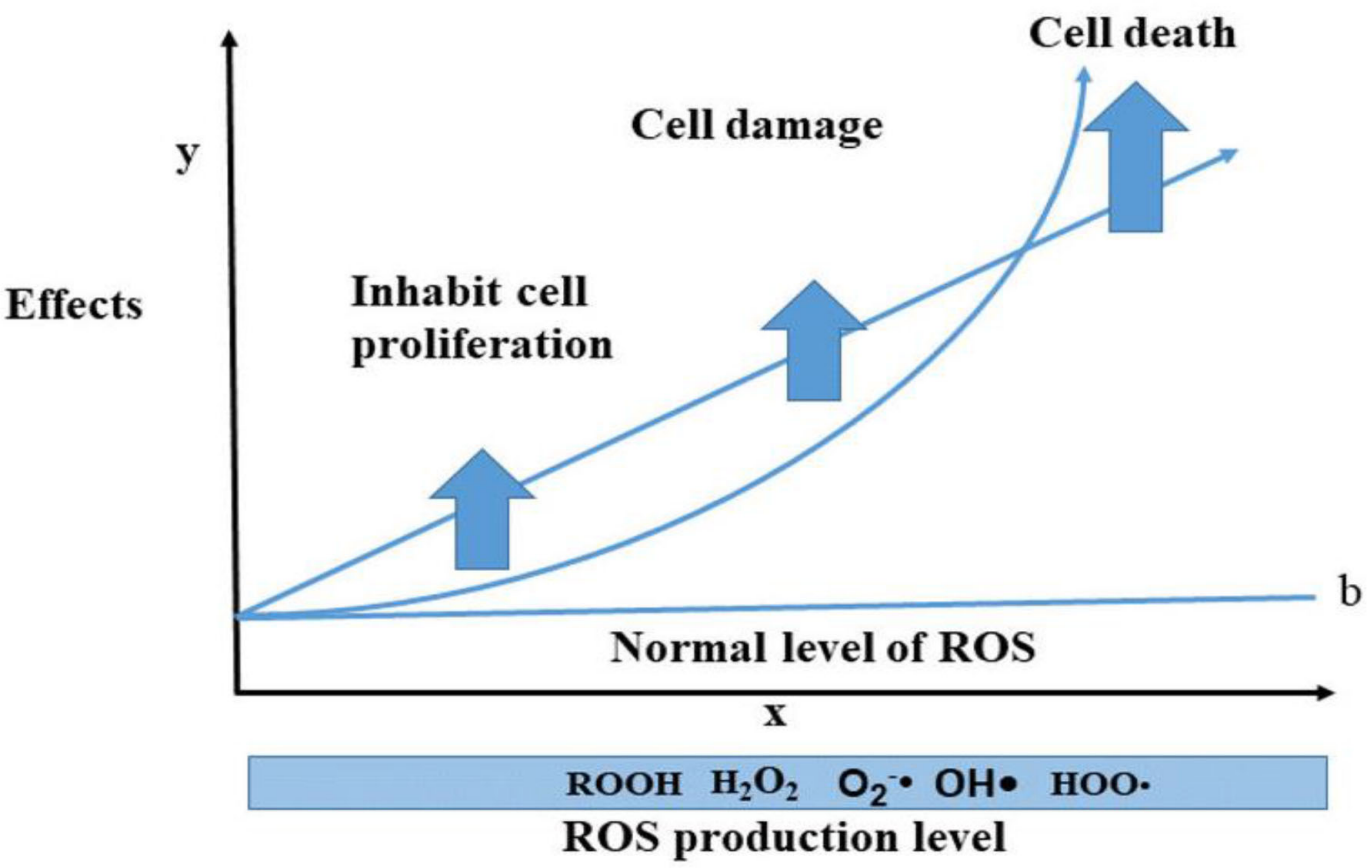
The trend of ROS levels and biological effects; the level of ROS increases cellular damages and cell death. This idea has been shown by two biological models such as linear and quadratic linear models (note: these two models are proposed by the authors for the ROS level and effects, considering the normal level for signaling pathways and cell proliferation).


In general, ROS are highly reactive molecules that are generated as byproducts of normal cellular metabolism. They play a crucial role in cellular signaling, but excessive ROS production can lead to oxidative stress and macromolecules damage, such as DNA, proteins, and lipids. ROS-induced oxidative stress causes accumulative damage over time and contributes to the aging process. Overall, understanding the role of ROS in cellular physiology and pathology is an active area of research, with important implications for aging, cancer, and other diseases. The level of ROS produced by internal and external sources cause biological effects such as cell proliferation or prevention of proliferation (CCA), organ, cell damage, and cell death. This article highlights the level of ROS and biological effects that follow from two mathematical models. The first one is linear model (1) and the second one is quadratic linear model (2). In both these models, we have considered the threshold or normal level of ROS (b) because there is an ROS level to regulate signaling pathways and normal cell proliferation in the body, while the damage happens when the level of ROS is above the threshold (123). In summary, **Figure 6** depicts the relationship between biological damage (effect) and ROS level, demonstrating how an increase in ROS level causes cancer cells death. These models share some similarities with mathematical dose–response curves used in radiobiology because of the way x-ray radiation and photodynamic treatment cause ROS and DNA damage (124).

(1) y=sx+b

where

y= damage or biological effects, x= ROS level, s= slope, and b= normal level of ROS.


(2) $$\text{y}={\text{sx}}^{2}+\text{b}$$

## 7 CCA following PDT

The role of PDT in the initiation of MC and CCA was first observed and studied with the emergence of second-generation PSs in PDT; most of them are in preclinical and clinical trials (**Table 1**) (12). Hence, molecular pathways of cell death in PDT and these PSs provide new insight for cancer therapy and modified the treatment strategy. As discussed in **Figure 1**, the PSs triggering cell death may have different cellular localizations. Since the range travel of ^1^O_2_ is only approximately 10–20 nm, the most damage and destruction is at the site of the PS during PDT.

**Table 1:** CCA *in vitro* and *in vivo* studies.

PSs	Type of cancer cell line	CCA	*In vitro/in vivo*	References
Methyl-aminolevulinate (MAL) (precursor for endogenous protoporphyrin IX)	HeLa	M	*In vitro*	(125)
TMPyP	HeLa and G361	M	*In vitro*	(126)
TMPyP4	A549	M	*In vitro*	(127)
TPFC	HeLa	M	*In vitro*	(128)
DTPP	A549	S	*In vitro*	(129)
DTPP	MCF-7	G0/G1	*In vitro*	(130)
Photocyanine	HepG2	G2/M	*in vitro*	(131)
Asymmetric glycophthalocyanine GPh3	HeLa	G2/M	*In vitro*	(132)
Zn(II)-phthalocyanine (ZnPc)	HeLa	M	*In vitro*	(133)
Malachite green (MG)	*Leishmania tropica* (*L. tropica*)	G0/G1	*In vitro*	(134)
Palmatine hydrochloride	OSCC	G0/G1	*In vitro*	(135)
Protoporphyrin IX (ALA)	OSCC	G2/M	*In vitro*	(136, 137)
3-hydroxypyridin-2-thione (SiPc-HDACi)	MCF-7/MDA-MB-231/HUVECs	G2/M	*In vitro*	(138)
(C086@HSA)	HeLa	S and G2/M	*In vitro/in vivo*	(139)
Fe3O4 nanoparticles (NPs) on Porphyrins(E-NPs)	AGS and RAW	G0/G1	*In vitro*	(140)
Curcumin	A549	G2/M	*In vitro*	(141)
Tetra-α-(4-carboxyphenoxy) phthalocyanine zinc (TαPcZn)	Bel-7402	S	*In vitro*	(142)
(ICG)	B16F10	G0/G1	*In vitro*	(143)
(AlPc) and (ZnPc)	A431	S and G2	*In vitro*	(144)
MPPa	MDA-MB- 231	S	*In vitro*	(145)
mTHPC	A-427, BHY, KYSE-70, RT-4, and SISO	G2/M	*In vitro*	(146)
Gemcitabine	RBE and QBC939	G1	*In vitro* and *in vivo*	(147)
Hypericin (HY)	ATL cells	G2/M	*In vitro*	(148)
(FA-LPNPs-VP-5-FU)	HCT116	G2/M and S	*In vitro*	(149)
Metformin	BCC	G0/G1	*In vitro*	(150)
Mitomycin C + protoporphyrin IX(ALA)	T24	G2/M	*In vitro*	(151)
Protoporphyrin IX (ALA)	PC-3	G0/G1	*In vitro*	(152)
Methylene blue (MB) and rutoside	A375	G0/G1	*In vitro*	(153)
AlPcS4Cl Gold NPs	A549	G1 and S	*In vitro*	(154)
Platinum(II) porphyrins (3-TPyP and 4-TPyP)	WM1366	No significant	*In vitro*	(155)
Pheophorbide a (Pa)	U87 MG, SK-OV-3, and HeLa	Only G0/G1 in U87 MG	*In vitro*	(156)

The CCA occurs at various phases as a result of the PDT-induced microtubule damage or distortion. Interestingly, mitotic block can happen in PDT, with or without PS photoinduction. For instance, the findings of Cenklová et al. has shown that α, β, χ, δ porphyrin-Tetrakis (1-methylpyridinium-4-yl) p-toluenesulfonateporphyrin (TMPyP) causes increased mitotic index and mitotic arrest in HeLa/G361 cells (126). Moreover, two separate studies by Csík et al. and Pizova et al. reported that TMPyP can cause DNA damage and protein interactions in the nucleosomes of G361 and MCF-7 cells, resulting in the alterations in DNA structure and gene expression (157, 158). Some porphyrins, by binding to tubulins, prevent their polymerization during combination therapy with PDT or single therapy (159). A study has shown the mechanism action of hypericin-based PDT that induces damage of microtubules and the mitotic spindle, resulting in CCA at the M phase (160). Dysfunction of some centrosomal proteins including AuroraA, ninein, TOG, and TACC3, which have an important role in microtubule arrangement, promotes apoptotic-like cell death (161). Mascaraque and colleagues reported that slight effects on spindle morphology lead to the distorting of microtubular of γ-tubulin and CCA in the mitotic phase in HeLa cells (125). The atomic force microscopy method by Jung et al. illustrated that chlorin under PDT disrupts the cytoskeleton in J82 bladder cancer cells and activation of apoptosis or programmable cell death (162). Furthermore, novel PS, DTPP leads to disruption of the cytoskeletal structure, cleavage of proteins, and CCA in the S phase in A549 cells following the mitochondria and lysosomes damaged by PDT-generated ROS (129, 163).

### 7.1 CCA at G2/M and M phase

The CCA at G2/M phase has been documented in some studies. Zheng et al. investigated the effects of 5,10,15,20-Tetrakis-(N-methyl-4-pyridyl) porphine (TMPyP4) in various concentrations on A549 cells *in vitro*. They found that TMPyP4, as a potential PS in PDT, arrested cells in M phase and prevented cell proliferation in doses more than 2 μM (127). Moreover, Barata and co-workers synthesized 5,10,15-tris (pentafluorophenyl) corrole (TPFC) as new PS, and their experiments on HeLa cells showed that TPFC has high photosensitizing efficiency in PDT and makes CCA in M phase via alterations on the mitotic spindle (128). In addition, the CCA, ROS production, and apoptosis effects of photocyanine in PDT had been examined by Shao et al. on HepG2 cells. They concluded that photocyanine mainly arrested the CC at G2/M phase and elevated the apoptosis rate via activation of caspase 3 (131). Another *in vitro* study showed that asymmetric glycophthalocyanine (GPh3) promotes the efficiency of PDT by arresting CC at G2/M stage; eventually, mitotic cell death happened in HeLa cells (132). Furthermore, it is reported that Zn (II)-phthalocyanine (ZnPc) in liposome structure has strong mitotic and metaphase blockage in neoplastic cells. Rello-Varona et al. represented that this phenomenon leads to apoptosis via PARP cleavage and Bax translocation to mitochondria in HeLa cells (133). Aru et al. synthesized silicon phthalocyanine substituted with 3-hydroxypyridin-2-thione (SiPc-HDACi) structure, which showed high potential of PS on MCF-7, HUVECs, and MDA-MB-231 cancer cells. Their findings indicated that SiPc-HDACi agent produces high ^1^O_2_ yield and can target nucleoli and CCA at G2/M in chemo-photodynamic therapy in all the three cell lines (138, 164). Another interesting study by He et al. showed that a new PS agent, C086-loaded human serum albumin (C086@HSA) NP, has anticancer effects in PDT. Not only they indicated that C086@HSA induces apoptosis and CCA at S and G2/M *in vitro* on HeLa cells, but also these results had been proven in 4T1 tumor-bearing mouse *in vivo* (139). Recently, the background of anticancer and PS effects of curcumin has attracted many researchers. However, Jiang et al. illustrated that curcumin in metal and encapsulation complex enhanced PDT efficiency. They found that encapsulated curcumin at concentration of 15 μM can arrest CC at G2/M phase in A549 cells (141, 145). In a study conducted by Lange et al., cell death pathways were compared under the porphyrin derivative 5,10,15,20-tetra(*m*-hydroxyphenyl) chlorin (mTHPC, temoporfin), as named Foscan in Europe (trade name) on various cell lines (A-427, BHY, KYSE-70, RT-4, and SISO) of head and neck cancers. Eventually, the experimental results showed that mTHPC-PDT causes DNA damage and CCA in the G2/M phase in all cell lines (146). Hypericin (HY) PS agent has shown excellent photosensitizing and anticancer effects against adult T-cell leukemia (ATL). HY-PDT treatment causes apoptosis induction and CCA at G2/M phase in leukemic cells through downregulation of Bcl-2 and expression of Bad, cytochrome c, and AIF (148). The Food and Drug Administration (FDA)-approved chemo-drug of 5-FU in incorporating a PS (verteporfin) with lipid–polymer hybrid nanoparticle delivery system (FA-LPNPs-VP-5-FU) complex has been used for colorectal cancer treatment in combination with radio-PDT. The findings of this study show that the FA-LPNPs-VP-5-FU nano-complex is strongly effective in inhibiting cancer cells proliferation and induces CCA in G2/M and S phases in HCT116 cells (149). Some studies have confirmed that mitomycin C causes CCA in G2/M phase in single treatment (165, 166). Furthermore a recent study by Nakayama et al. showed that this effect has been confirmed in combination with protoporphyrin IX (ALA) in PDT of prostate cancer (151).

### 7.2 CCA at G0/G1 phase

Cell cycle arrest at G0/G1 phase in PDT is common and has been reported in few studies. In a study by Ozlem-Caliskan et al., it was shown that malachite green (MG) has PS effects in PDT against ***Leishmania tropica*** disease. Their findings confirmed that MG at 6.25 μM dose in combination with 46.4 J/cm^2^ light at a wavelength of 550 nm exposure blocked cell cycle at G0/G1 phase (134). In 2019, Qi and colleagues reported the effects of palmatine hydrochloride (PaH) as alkaloid drug-mediated PDT on oral squamous cell carcinoma (OSCC). They confirmed that PaH-PDT increases the percentage of cells in the G0/G1 phase and decreases the CDK2 and cyclin E1 protein levels (135). However, it is shown that 5-aminolaevulinic acid (5-ALA) as a second generation of protoporphyrin IX PSs in PDT has no effect on CC but, as radiosensitizer (for gamma radiation), can stop oral squamous cell carcinoma at G2/M stage (136, 137). Furthermore, it is reported by Sengupta and collogues that Fe_3_O_4_ nanoparticles (NPs) on porphyrins has immune-protective effects and capable apoptosis via upregulations of p21 kinase by CCA at the G0/G1 stage in AGS and RAW cell lines (140, 142). In addition, the results of Radzi and co-workers demonstrated that applying indocyanine green (ICG) and PDT induces G0/G1 arrest and apoptosis in the B16F10 cells; moreover, PDT and hyperthermia had synergistic outcomes in the treatment of B16F10 cells (143). Furthermore, *in vitro* experiments of Gemcitabine and PDT showed that cholangiocarcinoma induces repressing cell viability, apoptosis, and eliciting of CCA in G1 phase via modulating cyclin D1 and caspase 3 cleavage. and prevents tumor growth *in vivo* (147). In addition, Mascaraque and colleagues evaluated the PS ability of metformin (Metf) on BCC cells. They revealed that Metf at 75 µM concentration arrests cells in G0/G1 phase and sensitize resistant cells to PDT through inhibition of the AMP-activated protein kinase (AMPK)/mammalian target of rapamycin (mTOR) pathway (150). The application of ALA and protoporphyrin IX (PpIX) are common in clinical use. High accumulation of PpIX can sensitize cancer cells to ALA-PDT. Nakayama et al. illustrated that PpIX accumulation in PC-3 cells can enhance ALA-PDT cytotoxicity by CCA at G0/G1 phase. Their data demonstrated that ALA-PDT would be an effective approach for cancer cells treatment and prevent cell growth (152). Khorsandi and co-workers have suggested a new regime treatment for PDT. They investigated the effect of rutoside an alkaloid agent in the combination therapy with methylene blue (MB) and PDT on A375 human melanoma cells. They found that MB-PDT and rutoside had better cytotoxic and antiproliferative effects on A375 cells and induced apoptosis and CCA at G0/G1 phase (153). Cho et al. assayed the PS effects of pheophorbide a (Pa) on three cell lines including U87 MG, SK-OV-3, and HeLa in PDT. Their findings showed that Pa has a strong effect on U87 MG cells. Furthermore, they found that Pa caused CCA in G0/G1 phase and DNA degradation, resulting in apoptotic cell death (156).

### 7.3 CCA at S phase

Wang et al. investigated the effects of 5-(4′-(2″ dicarboxymethylamino) acetamidophenyl)-10, 15, 20-triphenylporphyrin (DTPP) as a new PS in PDT on A549 cells. Their data have shown that DTPP arrested CC at S phase, leading to cytoskeleton collapse. Moreover, they investigated DTPP on MCF-7 cells and concluded that DTPP arrests growth and microtubule alteration of MCF-7 cells at 4 μg/ml concentration (129, 130). Moreover, tetra-(4-carboxyphenoxy) phthalocyanine zinc (TαPcZn), a novel hydrophilic/lipophilic PS designed and synthesized by Xia et al. in 2011, was utilized to mediate PDT on Bel-7402 cells, and their results indicated that TαPcZn downregulated bcl-2 and fas genes and arrested CC at S phase and proliferation inhibition significantly (142). In 2021, Dias et al. evaluated the PS effects of metallated phthalocyanine forms of aluminum phthalocyanine (AlPC) and zinc phthalocyanine (ZnPC) and their tetrasulfonated derivatives of AlPCS4 and ZnPCS4 on A431 cells. Their findings illustrated that ZnPc, AlPc, and AlPcS4 photosensitized cells at LC50 values of 0.13, 0.04, and 0.81 μM, respectively, after 24 h PDT. However, ZnPcS4 did not induce notable phototoxicity. Moreover, their results proved that CCA happened in the S phase (ZnPc, AlPc, and AlPcS4) and G2 phase (ZnPc and AlPc) (144). Liang and co-workers showed that pyropheophorbide-a methyl ester (MPPa), a derivative of chlorophyll, has PS effects in PDT on MDA-MB-231 cells and induces cell death via apoptosis and CCA at S phase. Additionally, they verified that following PDT therapy, Chk2 and P21 expression increased, whereas cyclin D1 expression was dramatically reduced (145). Recently, nanoparticles, as carrier to deliver PS drugs, have attracted researchers in this area. For instance, Crous and Abrahamse have reported that aluminum (III) phthalocyanine chloride tetra-sulfonate (AlPcS_4_Cl) conjugated on gold nanoparticle in PDT prevents lung cancer metastasis. Their study has shown that AlPcS_4_Cl conjugated on gold NPs can prevent lung cancer metastasis and invasion via CCA in G1/S phases and cell death (154). A study by Couto et al. reported that although platinum (II) porphyrins is a good PS, it has no effect on cell cycle on WM1366 cells. Their findings illustrated that although an overexpression of the P21 gene (involved in the CC) was observed in the qRT-PCR, they did not observe a significant stop in the G0/G1, S, and G2/M cycle (155).

## 8 The potential of PDT to temporarily stop or synchronize cancer cells

CCA and cell synchronizing refer to the process of temporarily stopping or synchronizing the growth and division of cells. This can be used in cancer therapy to improve the effectiveness of treatments and reduce their toxicity on normal healthy cells. By targeting cancer cells during specific phases of the CC, such as during DNA replication or cell division, therapies can be optimized for individual patients and may help to reduce the development of treatment resistance. Additionally, studying the relationship between the CC and cancer biology can provide insights into new treatment approaches and therapies. There are some potential advantages of cell synchronization in cancer therapy:

1. Increased effectiveness: cell synchronization can be used to coordinate the timing of cancer treatments with the CC, maximizing the effectiveness of CT, RT and PDT.
2. Reduced toxicity: by targeting cancer cells during specific phases of the CC, cell synchronization can help reduce the toxicity of cancer treatments on healthy cells.
3. Personalized treatment: cell synchronization can help to tailor cancer treatments in individual patients, based on their unique CC characteristics.
4. Improved outcomes: by optimizing the timing of cancer treatments, cell synchronization may help to improve patient outcomes and increase survival rates.
5. Better understanding of cancer biology: studying the CC and its relationship to cancer can provide insights into the underlying biology of the disease, leading to new treatment approaches and therapies.
6. Reduced treatment resistance: by targeting cancer cells during specific phases of the CC, cell synchronization may help to reduce the development of treatment resistance, which is a major challenge in cancer therapy.

**Figure 7:**
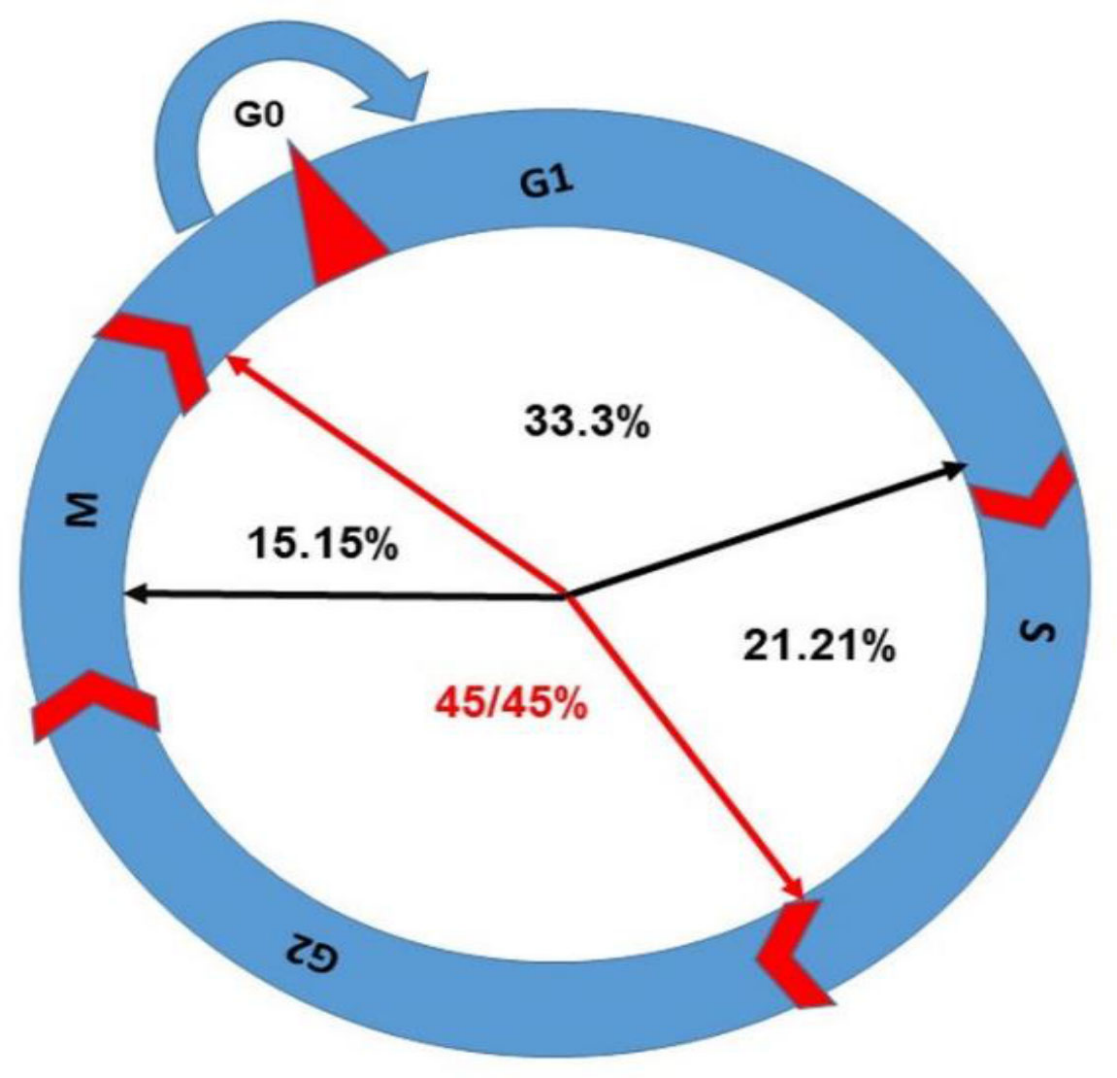
The percentage of CCA in different phases has been shown in this figure; G2/M arrest is the highest with 45.45% as per reports discussed in this review.

**Figure 8:**
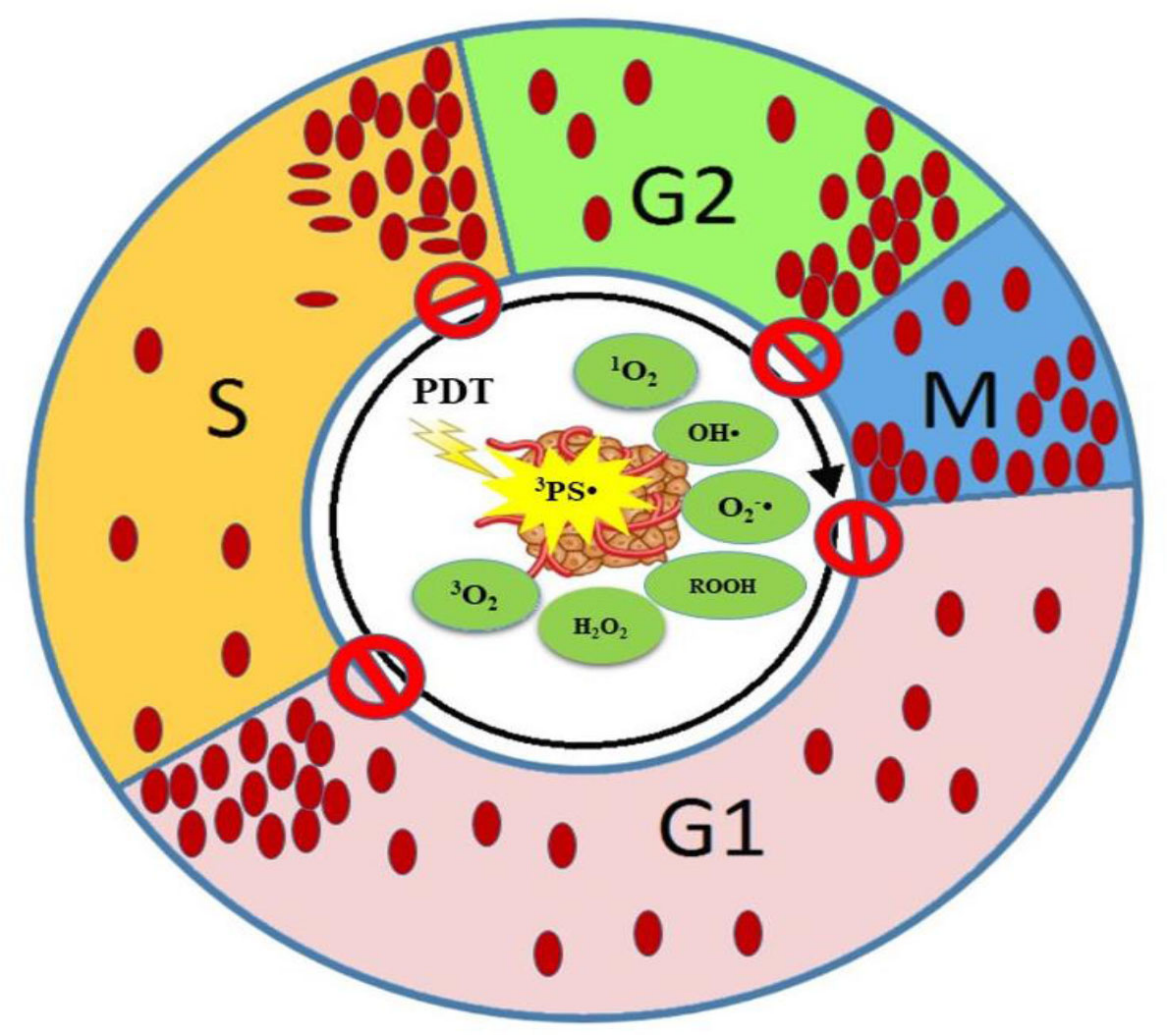
Schematic representation of CCA by PDT at different phases of the CC. ROS cause cellular and DNA damage resulting in CCA at various phases.

However, from the results in **Table 1**, it seems that the mechanism of actions of PDT on CCA depends on the type of cell line, PS agent, and ROS level. Consequently, the organelles and DNA damages lead to CCA. The CCA prevents cell progression and synchronization. We come to the conclusion that even though CCA has been documented in phases like G0/G1, S, and G2/M, the highest percentage of CCA has been reported in G2/M phase under PDT. DNA is considered as the sensitive target to ROS action directly and indirectly. From **Table 1**, among 33 studies that have been investigated on CCA in PDT, approximately 45.45% of them have reported that the G2/M phase is the most sensitive phase of CC to be arrested for cancer therapeutic application (**Figures 7**, **8**). In summary, PDT can arrest CCA at different stages of CC, which can act as a strong cancer cells progression barrier, and this mechanism could help to improve treatment efficacy via different doses of PS and light irradiation.

## 9 Conclusion and perspectives

The standardization of PDT protocol remains one of the critical challenges to resolve. To overcome this challenge, the biological response of PDT needs to be investigated. CCA is common in apoptosis induced by PDT; it seems that some of the PS has their own special action without PDT. This review highlights the opportunities to develop PDT standardization protocol and combinatorial treatments. Authors introduced two mathematical models of ROS level and its effects in cells. Furthermore, effects of ROS level on oxidative stress, the mitotic checkpoint, and delaying or arresting CC progression have been reviewed. We have suggested new strategies that PDT could be used alone or in combination with immunotherapy, RT, or chemo-therapeutic drugs in cancer therapy because the mechanism of action of PDT is similar to chemo-radiotherapy in some ways. However, they can have synergistic effects on cancer treatment. One of the important issues in combination therapy is the decision on the prioritization of the modalities in order to achieve high treatment efficiency. Hence, understanding the CC control in cancer cells could open therapeutic opportunities to improve treatment outcomes.

The ideal PDT protocol should provide some requirements including the following: first is predicting tumor sensitivity to PDT; second is the choice of an appropriate PS with low toxicity and high photochemical activity on CCA; third, targeting and concentration of PS and irradiation dose should be tailored to the tumor; fourth, the evaluation of oxygen level in the tumor microenvironment; fifth, the reparation of PS and radiation dose after detecting CCA can be more effective; and finally, PDT should activate the immune system.

To sum up, standardization is one of the challenges in PDT. There is currently no standardized protocol for PDT, which can lead to variability in treatment outcomes and hinder its widespread adoption. Developing standardized guidelines for patient selection, dosing, and treatment parameters is essential for improving the consistency and reproducibility of PDT. However, some research has suggested that targeting cancer cells during specific phases of the CC may enhance the effectiveness of PDT. For example, targeting cancer cells in the S phase (DNA synthesis phase) may increase the amount of PS uptake and ROS generation, leading to more efficient cell death pathways. However, further research is needed to understand the potential benefits of synchronizing cancer cells in PDT. In addition, as a suggestion, most PS drug and nano-photosensitizers have the potential as radiosensitizers, so they can be used in combination with PDT and RT in cancer therapy. This suggestion helps in highly effective treatment and prevention of cell proliferation via CCA. Furthermore, stopping CC with repetition PDT regime promotes to more cell damage and cell death. From a prospective view, the CC studies *in vitro* can be confirmed by *in vivo* testing with repeated PS administration and irradiation to confirm better treatment efficiency. Moreover, as we reviewed, CCA in specific phases can be a specific feature to determine cell line and PS drug, so this feature could be considered in tailoring therapy in PDT.

## Author contributions

Conceptualization, KM and BG. Writing—original draft preparation, KM. Writing—review and editing, BG and HA. Supervision, BG and HA. These authors contributed equally to this work. All authors contributed to the article and approved the submitted version.
